# Inactivation of the DNA Repair Genes *mutS, mutL* or the Anti-Recombination Gene *mutS2* Leads to Activation of Vitamin B_1_ Biosynthesis Genes

**DOI:** 10.1371/journal.pone.0019053

**Published:** 2011-04-28

**Authors:** Kenji Fukui, Taisuke Wakamatsu, Yoshihiro Agari, Ryoji Masui, Seiki Kuramitsu

**Affiliations:** 1 RIKEN SPring-8 Center, Harima Institute, Kouto, Sayo-gun, Hyogo, Japan; 2 Graduate School of Frontier Biosciences, Osaka University, Yamadaoka, Suita, Osaka, Japan; 3 Department of Biological Sciences, Graduate School of Science, Osaka University, Machikaneyama-cho, Toyonaka, Osaka, Japan; St. Georges University of London, United Kingdom

## Abstract

Oxidative stress generates harmful reactive oxygen species (ROS) that attack biomolecules including DNA. In living cells, there are several mechanisms for detoxifying ROS and repairing oxidatively-damaged DNA. In this study, transcriptomic analyses clarified that disruption of DNA repair genes *mutS* and *mutL*, or the anti-recombination gene *mutS2*, in *Thermus thermophilus* HB8, induces the biosynthesis pathway for vitamin B_1_, which can serve as an ROS scavenger. In addition, disruption of *mutS*, *mutL*, or *mutS2* resulted in an increased rate of oxidative stress-induced mutagenesis. Co-immunoprecipitation and pull-down experiments revealed previously-unknown interactions of MutS2 with MutS and MutL, indicating that these proteins cooperatively participate in the repair of oxidatively damaged DNA. These results suggested that bacterial cells sense the accumulation of oxidative DNA damage or absence of DNA repair activity, and signal the information to the transcriptional regulation machinery for an ROS-detoxifying system.

## Introduction

In living cells, extracellular oxidative stress and intracellular redox reactions of aerobic metabolism generate reactive oxygen species (ROS) that are harmful for biomolecules such as proteins, lipids, carbohydrates, and DNAs [1]. A radical attack on the bases in DNA produces oxidized bases such as thymine glycol, 2,6-diamino-4-hydroxy-5-formamidopyrimidine, 5-hydroxymethyluracil, 8-oxoguanine (8OG), and 5-formyluracil [2], [3]. Through DNA replication or error-prone repair events, the modification of bases can result in the alteration of genetic information because an oxidized base can form stable hydrogen bonds with multiple partners. For example, 8OG and 5-formyluracil can pair not only with cytosine and adenine but also with adenine and guanine or cytosine, respectively [4], [5], [6]. Although such mutagenesis can be a driving force for evolution to survive a specific environment, cells, under normal conditions, need to avoid frequent alteration of their genome. Cells are equipped with both protection mechanisms against ROS and repair mechanisms for damaged DNA to prevent cell death or to suppress the rate of mutagenesis.

As protection mechanisms against ROS, several enzymatic and non-enzymatic ones are known. Catalases, superoxide dismutases, and peroxidases catalyze the reduction of superoxide or hydrogen peroxide [7]. Glutathione, vitamin E, vitamin C, vitamin B_6_, β-carotene, and bilirubin have been found to detoxify various kinds of ROS [8], [9], [10].

As a repair mechanism, it has been well established that base-excision repair system removes oxidatively damaged bases from DNA [3], [4]. In this repair system, a specific DNA glycosylase (MutM and OGG1 in bacteria and humans, respectively) removes the 8OG residue from an 8OG:cytosine pair [11]. An unrepaired 8OG:cytosine pair can be converted to an 8OG:adenine pair through DNA replication. Removal of 8OG from the 8OG:adenine pair is an error-prone process that fixes the GC-TA transversion mutagenesis. Then, another DNA glycosylase (MutY and MYH in bacteria and humans, respectively) excises the adenine residue from an 8OG:adenine pair to re-generate 8OG:cytosine pair, which is a substrate for MutM glycosylase [12]. Furthermore, it has also been suggested that DNA mismatch repair (MMR) system plays the same role as MutY in the removal of the adenine residue from an 8OG:adenine pair in a DNA replication-dependent manner [13], [14], [15], [16]. MMR recognizes an 8OG:adenine pair as a mismatched base pair, and removes the adenine residue in the newly-synthesized strand.

Interestingly, it has been reported that MutS and MutL, the key enzymes in MMR, are limiters of the stationary phase-induced/adaptive mutagenesis in *Escherichia coli* and *Saccharomyces cerevisiae*, respectively [17], [18]. Since it has also been reported that the main driving force of the stationary phase-induced mutagenesis may be oxidative stress [19], it is expected that MutS and MutL can function in the repair of oxidative DNA damage during the stationary phase. Previously known MMR depends on replicative DNA polymerases such as DNA polymerase III or DNA polymerase δ. However, those replicative DNA polymerases are expected to be inactive in stationary phase cells, where replication events merely occur. In fact, in *Thermus thermophilus* HB8, the expression of β-subunit of DNA polymerase III gene is potently suppressed in stationary phase (GEO accession number: GSE19839). Therefore, it could be speculated that MutS and MutL are involved in the repair of oxidative DNA damages in a different manner from the known MMR system. The cooperation of MMR proteins with an error-prone DNA polymerase or base-excision repair system has been discussed [19], [20].

**Figure 1:**
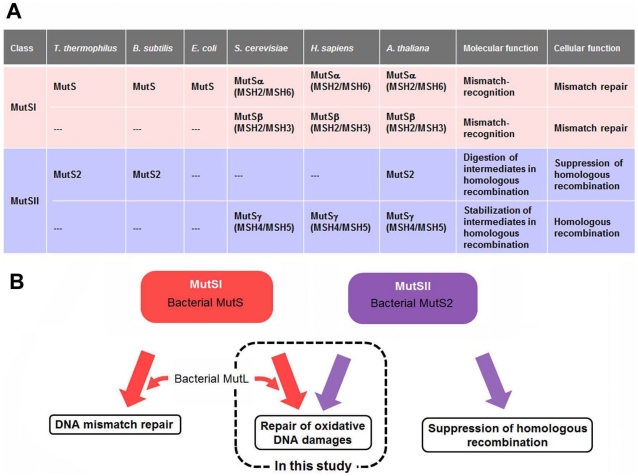
MutS family proteins. (A) MutS family proteins can be classified into MutSI and MutSII subfamilies. MutSI includes bacterial MutS, and eukaryotic MutSα and MutSβ, which are involved in MMR [69], [72]. MutSII includes eukaryotic MutSγ, and bacterial MutS2, which are involved in promotion and suppression of homologous recombination, respectively. Bacterial MutS and MutS2 are homodimeric protein, while eukaryotic MutSα, MutSβ, and MutSγ are heterodimeric proteins comprised of MSH2/MSH6, MSH2/MSH3, and MSH4/MSH5, respectively. (B) Divergence in functions of bacterial MutS and MutS2. In this study, it was suggested that MutS and MutS2 cooperatively participate in repair of oxidative DNA damages.

We have been studying DNA repair enzymes from *Thermus thermophilus* HB8, which include MutS, MutL, and MutS2. Bacterial MutS and MutL play central roles in MMR [21], [22], [23], [24] in a similar manner to well-characterized eukaryotic homologues [25], [26]. MutS recognizes mismatched base pairs and MutL is thought to interact with a MutS-mismatch complex to initiate excision of the error-containing strand. Bacterial MutS2 is a paralogue of MutS and is not involved in MMR but in the suppression of homologous recombination [27], [28], [29] (Fig. 1A). However, it is suggested that *Helicobacter pylori* MutS2 participates not only in the suppression of homologous recombination but also in the repair of oxidative DNA damage [30]. We have reported that recombinant *T. thermophilus* MutS2 interacts with MutL, although the biological significance of this interaction remains unknown [31]. In addition, high similarity in dimerization domain between bacterial MutS and MutS2 raises the possibility that MutS may interact with MutS2 [31].

In this study, to assess the cooperative involvement of MutS, MutL, and MutS2 in DNA repair, interactions of MutS2 with MutS and MutL were examined by co-immuno precipitation assay. The results clearly showed *in vivo* interactions between them. We also performed transcriptomic analyses of *T. thermophilus* HB8 strain lacking *mutS*, *mutL*, or *mutS2*. As a result, it was confirmed that vitamin B_1_ (thiamine) biosynthesis was induced in all three disruptant strains and that vitamin B_1_ can act as an antioxidant against ROS. A significant effect of disruption of *mutS*, *mutL*, or *mutS2* on the rate of oxidative stress-induced mutagenesis was also demonstrated. These data suggest that *mutS*, *mutL*, and *mutS2* cooperatively function in the repair of oxidative DNA damage (Fig. 1B) and that the loss of this function leads to the induction of biosynthesis of vitamin B_1_ as an ROS scavenger.

## Methods

### Culture conditions for *T. thermophilus* HB8

*T. thermophilus* HB8 [32], [33] was grown at 70°C in TR medium: 0.4% tryptone (Difco Laboratories, Detroit, MI), 0.2% yeast extract (Oriental Yeast, Tokyo, Japan), and 0.1% NaCl (pH 7.5) (adjusted with NaOH). To make plates, 1.5% gellan gum (Wako, Osaka, Japan), 1.5 mM CaCl_2_, and 1.5 mM MgCl_2_ were added to TR medium (metals are necessary to solidify the gellan gum).

### *T. thermophilus* HB8 strains

*T. thermophilus* HB8 strains lacking *mutM* (Δ*mutM*) and *ttha0675* (Δ*ttha0675*) were constructed by substituting the target gene with the thermostable kanamycin-resistance gene, *HTK* [34], through homologous recombination as previously described [35], [36]. The plasmids used for gene disruption were derivatives of the pGEM-T Easy vector (Promega Co., Madison, WI), constructed by inserting *HTK* flanked by approximately 500-bp upstream and downstream sequences of each gene. The plasmids were transformed into *T. thermophilus* HB8 cells as described previously [35]. Gene disruptions were confirmed by PCR amplification using the isolated genomic DNAs as templates.

A *T. thermophilus* HB8 strain lacking *mutS* (Δ*mutS*), *mutL* (Δ*mutL*), or *mutS2* (Δ*mutS2*) was generated as described previously [23], [29]. Since *mutL* locus is in the upstream of *mutS* locus in the same operon, the disruption of *mutL* was performed by inserting the reversed *HTK* cassette.

### Co-immunoprecipitation and Western blotting

*T. thermophilus* HB8 cells were grown in 50 ml of TR medium at 70°C for 15 h and then harvested by centrifugation. Cells were lysed in a buffer comprising 50 mM Tris-HCl (pH 7.5) and 30 mM NaCl, and bovine pancreas DNase I (Takara, Shiga, Japan) was added to 0.5 mg/ml. After incubation at 37°C for 30 min, the lysate was subjected to immunoprecipitation. Immunoprecipitation was performed with Dynabeads-Protein A conjugates (Veritas, Tokyo, Japan) according to the manufacturer's instructions. Ten µg of polyclonal rabbit anti-MutS (Oriental Yeast Co., Tokyo, Japan), -MutL (MBL Co., Nagoya, Japan), or -MutS2 (Oriental Yeast Co.) antibody (Oriental Yeast Co., Tokyo, Japan) was used for binding to 50 µl of Dynabeads-Protein A conjugate.

The immunoprecipitates were resolved on 12% acrylamide gels and electroblotted onto PVDF membrane (Millipore, Milford, MA). The membrane was incubated in a blocking solution comprising 20 mM Tris-HCl (pH 7.5), 500 mM NaCl, and 3% gelatin (Bio-Rad Laboratories, Hercules, CA) for 30 min at room temperature. After washing with 20 mM Tris-HCl (pH 7.5) containing 500 mM NaCl, and 0.05% Tween-20 (Bio-Rad Laboratories), the membrane was immersed into the same buffer containing rabbit anti-MutL, -MutS, or -MutS2 antibody and then incubated for 12 h at room temperature. After washing in 20 mM Tris-HCl (pH 7.5) containing 500 mM NaCl, and 0.05% Tween-20, the membrane was reacted with Protein A-horseradish peroxidase conjugate (Bio-Rad Laboratories) in a buffer comprising 20 mM Tris-HCl (pH 7.5), 500 mM NaCl, 0.05% Tween-20, and 1% gelatin for 2 h at room temperature. The membrane was washed twice in 20 mM Tris-HCl (pH 7.5), 500 mM NaCl and 0.05% Tween-20, and then reacted with 4-chloro-1-naphthol (Bio-Rad Laboratories) in HRP color development buffer (Bio-Rad Laboratories) for 30 min at room temperature. The staining was stopped by washing in deionized water.

### Preparation of His_6_-tagged MutS2

The *Nde*I-*Bgl*II region of the pT7Blue/*mutS2* plasmid [31] was ligated into the *Nde*I-*Bam*HI site of the pET-15b vector (Novagen, Madison, WI) to yield the pET-15b/*mutS2* plasmid for expression of His_6_-tagged MutS2. *E. coli* Rosetta-gami(DE3) (Novagen) was transformed with pET-15b/*mutS2* and cultured in 1.5 l of YT medium containing 50 µg/ml ampicillin at 37°C. When the density of cultures reached 4×10^8^ cells/ml, isopropyl-β-D-thiogalactopyranoside (Wako) was added to 1 mM. The cells were grown at 37°C for 4 h after induction and then harvested by centrifugation. The cells were lysed by sonication in buffer I (20 mM Tris-HCl (pH 7.5) and 500 mM NaCl) and then heated to 70°C for 10 min. After centrifugation at 48,000 × *g* for 60 min, the supernatant was loaded onto 10 ml of Talon Resin (Clontech, Palo Alto, CA) pre-equilibrated with buffer I. The resin was washed with 200 ml of buffer I containing 10 mM imidazole and then eluted with a 200 ml gradient of 10–500 mM imidazole in buffer I. The fraction containing His_6_-tagged MutS2 was loaded onto a HiLoad 16/60 Superdex 200 pg column (GE Healthcare Biosciences, Uppsala, Sweden) pre-equilibrated with a buffer comprising 20 mM Tris-HCl (pH 8.0) and 200 mM NaCl, and eluted with the same buffer. The eluted His_6_-tagged MutS2 was concentrated to 20 µM using a Vivaspin concentrator (Vivascience, Hanover, Germany). Peptide mass fingerprinting [37] revealed that the purified protein was His_6_-tagged MutS2. The protein concentration was determined on the basis of the absorbance at 278 nm using the molar extinction coefficient of 21,945 M^−1^cm^−1^ calculated by the previously described procedure [38].

### Pull-down assay

*T. thermophilus* HB8 cells were grown in 50 ml of TR medium at 70°C for 15 h and then harvested by centrifugation. Cells were lysed in 1 ml of buffer I, and then subjected to pull-down assay.

Two hundred µl of 20 µM His_6_-tagged MutS2 was loaded onto 200 µl of Talon resin pre-equilibrated with buffer I in a microtube. The resin was washed twice with 1 ml of buffer I. One ml of cell lysates were loaded onto the His_6_-tagged MutS2-bound Talon resin, followed by incubation in the presence or absence of 5 mM ATP or ADP and 5 mM MgCl_2_ for 30 min at room temperature. The resin was washed four times with 1 ml of buffer II (20 mM Tris-HCl (pH 7.5), 100 mM KCl, 0 or 5 mM ATP or ADP, 5 mM MgCl_2_, 1 mM dithiothreitol (DTT), and 0.1% BSA) containing 10 mM imidazole. His_6_-tagged MutS2 was eluted with buffer II containing 100 mM imidazole. The eluted fractions were subjected to Western blotting analysis using anti-MutS or -MutL antibody.

### DNA microarray

DNA microarray analyses were performed as described previously [39], [40]. Wild-type, Δ*mutS*, Δ*mutL*, and Δ*mutS2 T. thermophilus* HB8 strains were cultured in TR broth at 70°C until the OD_600_ reached ∼0.5, and then cells were harvested from 50 ml of the culture. Total RNA was extracted from each strain, and then cDNAs were synthesized by SuperScript II (Invitrogen, Carlsbad, CA) and 6-base random primers (Invitrogen) in the presence of the RNase inhibitor SUPERase (Ambion, Austin, TX), fragmented by DNase I (GE Healthcare), and labeled with biotin by the GeneChip DNA labeling reagent (Affymetrix, Santa Clara, CA). The labeled cDNA fragments were hybridized with a TTHB8401a520105F GeneChip (Affymetrix) that contained probe sets of 25-mer oligonucleotides for 2238 ORFs and 1096 intergenic regions. After washing and staining with streptavidin-phycoerythrin (Invitrogen) by GeneChip Fluidics Station 450XP (Affymetrix), the array was scanned by a GeneChip Scanner 3000 (Affymetrix).

The expression intensities of the 2238 ORFs for three lots of the independently cultured wild type, Δ*mutS*, Δ*mutL*, and Δ*mutS2* strains were evaluated by GeneChip Operating Software version 1.2 (Affymetrix) as described previously [40] using the definition of platform GPL4902 (NCBI Gene Expression Omnibus (GEO; http://www.ncbi.nlm.nih.gov/geo/)). The data set analyses were performed on Subio platform version 1.6 (Subio Inc., Tokyo, Japan). First, measurement values less than 1 were transformed to 1. Second, the data were transformed into logarithms and normalized through global normalization (normalized as to the median). Third, the data were normalized as to the mean of the wild-type data. The microarray data discussed in this study are MIAME compliant and have been deposited in the GEO and are accessible through GEO Series Accession No. GSE22567.

### Reverse transcription (RT)-PCR

RT-PCR was performed as described previously [41] using a PrimeScript RT-PCR kit (Takara). The primers (BEX Co., Tokyo, Japan) used are listed in Supplementary Table S1.

### Examination of sensitivity to H_2_O_2_

The wild-type, Δ*mutS*, Δ*mutL*, Δ*mutS2,* Δ*mutM,* and Δ*ttha0675 T. thermophilus* HB8 strains were grown in 3 ml of TR medium for 16 h. The precultured cells were suspended in 3 ml of TR medium and cultured to 1×10^8^ cells/ml. Forty-five µl of each culture was mixed with 5 µl of 0, 30, 60, 120, 150, 300, or 450 mM H_2_O_2_ in the presence of various concentrations of vitamin B_1_ hydrochloride (Wako). The H_2_O_2_ solutions were added to the cell culture 5 min after the addition of vitamin B_1_ hydrochloride. The concentrations of vitamin B_1_ are indicated in the figure or figure legend. After incubation at 70°C for 2 min, 2 µl of each mixture was spotted onto a TR plate, followed by incubation at 70°C for 16 h.

In order to test the effect of 10 mM H_2_O_2_ on the growth of *T. thermophilus* HB8 cells, the following experiment was performed. The wild-type, Δ*mutS*, Δ*mutL*, Δ*mutS2,* and Δ*mutM* strains were grown in 3 ml of TR medium for 16 h. The precultured cells were suspended in 5 ml of TR medium to an OD_600_ value of 0.10. After incubation at 70°C for 3 h, 50 µl of 0 or 1 M H_2_O_2_ was added. The cell cultures were incubated at 70°C for 4 h. The growth of the cells was monitored by measuring OD_600_.

### Estimation of the mutation frequency under oxidative stress

The mutation frequency of *T. thermophilus* HB8 was estimated based on the frequency of streptomycin-resistant mutants measured by means of the modified Luria-Delbruck fluctuation test [42] as described previously [23]. Streptomycin is an antibiotic agent that binds to the 30S ribosomal subunit and interferes the initial selection and proof-reading steps of translation [43]. A single amino acid substitution in streptomycin-binding site of the ribosomal protein S12 or a point mutation in 16S rRNA can lead to the acquisition of streptomycin resistance [44]. The wild-type, Δ*mutS*, Δ*mutL*, and Δ*mutS2 T. thermophilus* HB8 strains were cultured in 3 ml of TR medium at 70°C for 24 h. The cultures were diluted 1∶60 with 3 ml of TR medium and then shaken at 70°C for 6 h (∼1×10^9^ cells/ml). The cultures were mixed with 30 µl of 0 or 1 M H_2_O_2_ and then incubated at 70°C for 30 min. The 1 ml of each culture was spread on a plate containing 50 µg/ml streptomycin. The same cultures were diluted 1∶10^5^ with TR medium and 100 µl of each diluted culture was spread on a drug-free plate. The plates were incubated at 70°C for 16 h. The frequency of streptomycin-resistant mutants per 10^8^ cells was calculated from the numbers of colonies formed on the streptomycin-containing and drug-free plates.

## Results

### MutS2 interacted with MutS and MutL

**Figure 2:**
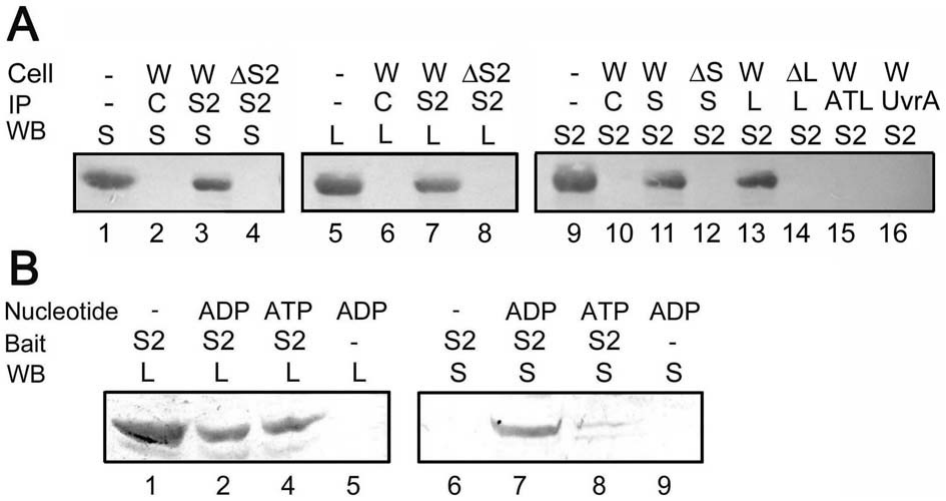
Interactions between MutS and MutS2, and MutL and MutS2. (A) Co-immunoprecipitation. “Cell” indicates strains used for immunoprecipitation, where W, ΔS, ΔL, and ΔS2 represent the wild-type, Δ*mutS*, Δ*mutL*, and Δ*mutS2* strains, respectively. IP and WB indicate antibodies used for immunoprecipitation and Western blotting, respectively. S, L, S2, ATL, and UvrA indicate anti-MutS, -MutL, -MutS2, -ATL protein, and -UvrA antibodies, respectively. C indicates pre-immune antibody. (B) Pull-down assay. S2 indicates recombinant His_6_-tagged MutS2 used as a bait protein. WB indicates antibodies used for Western blotting. L and S indicate anti-MutL and -MutS antibodies used for Western blotting.

Our previous result exhibited *in vitro* interaction of *T. thermophilus* MutS2 with MutL [31]. In addition, comparison of amino acid sequences of *T. thermophilus* MutS and MutS2 showed that MutS2 contains a region corresponding to the dimerization domain of MutS [31], which implies the interaction between MutS2 and MutS. To address this issue, we examined the *in vivo* interaction of MutS2 with MutS or MutL. As shown in Fig. 2A, MutS and MutL were co-immunoprecipitated with MutS2 by using anti-MutS2 antibody. It was also confirmed that MutS2 was co-immunoprecipitated with MutS or MutL by using anti-MutS or -MutL antibody. The co-immunoprecipitation was not observed when Δ*mutS*, Δ*mutL*, and Δ*mutS2* strains were used (Fig. 2A, lines 4, 8, 12, and 14). In addition, MutS2 was not co-immunoprecipitated with other unrelated DNA-binding proteins, *T. thermophilus* UvrA, the nucleotide-excision repair enzyme [45], or Alkyltransferase-like (ATL) protein, the *O* ^6^-methylguanine repair enzyme [46] when anti-UvrA or -ATL protein antibody was used (Fig. 2A). These results indicate that MutS and MutL form complexes with MutS2 in the cell.

Pull-down assays involving recombinant His_6_-tagged MutS2 as a bait protein also support the interactions of MutS2 with MutS and MutL (Fig. 2B). Interestingly, MutS was not pulled down by MutS2 under the condition without a pre-incubation of MutS2 with an adenine nucleotide, and the pre-incubation of MutS2 with ADP greatly enhanced the interaction between MutS2 and MutS. In contrast, the interaction between MutS2 and MutL seemed to be only slightly reduced by the addition of adenine nucleotides to the assay system. The effect of adenine nucleotides on the interaction properties of MutS is quite reasonable because it is known that MutS family proteins including MutS2 change their conformations and functions in response to binding of adenine nucleotides [29], [47], [48], [49].

### Disruption of *mutS*, *mutL*, or *mutS2* activated vitamin B_1_ biosynthesis

**Figure 3:**
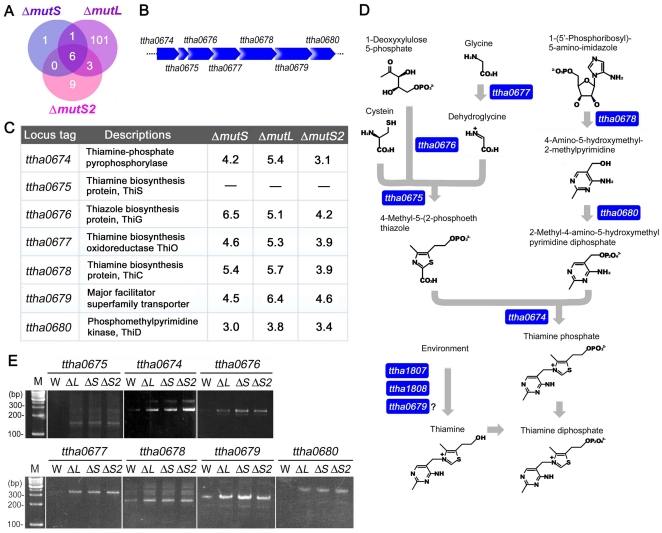
DNA microarray analyses of the Δ*mutS*, Δ*mutL*, and Δ*mutS2* strains. (A) A Venn diagram shows the up-regulated genes in the three disruptants. (B) A schematic representation of vitamin B_1_ biosynthesis operon in *T. thermophilus* HB8. (C) Vitamin B_1_ biosynthesis genes were up-regulated in all three disruptants. Expression in the disruptants relative to that in the wild-type strain is indicated, where the *P*-values are less than 0.00076. The respective *P*-values are listed in Supplementary Tables S2, S3, S4. The values for *ttha0675* were determined by using the definition in the platform GPL9209 (GEO accession number: GPL9209). (D) A predicted model of biosynthesis pathway of thiamine diphosphate in *T. thermophilus* HB8. Pyrimidine and thiazole moieties are synthesized separately and then combined to form thiamine phosphate. (E) RT-PCR confirmed the up-regulation of vitamin B_1_ biosynthesis genes in each disruptant. DNA fragments were amplified using total RNAs as templates, and then subjected to agarose gel electrophoresis. M, W, ΔL, ΔS, and ΔS2 represent the 100-bp ladder DNA size marker, and the wild-type, Δ*mutS*, Δ*mutL*, and Δ*mutS2* strains, respectively. Primers were designed to amplify 161-, 289-, 295-, 320-, 231-, 290-, and 365-bp DNA fragments from the cDNAs of *ttha0675*, *ttha0674*, *ttha0676*, *ttha0677*, *ttha0678*, *ttha0679*, and *ttha0680*, respectively.

In order to well define the cellular functions of *mutS*, *mutL*, and *mutS2*, we analyzed transcription of the whole genome in *T. thermophilus* HB8 cells lacking *mutS* (Δ*mutS*), *mutL* (Δ*mutL*), and *mutS2* (Δ*mutS2*) during the exponential growth phase by DNA microarray. When compared with wild-type cells using the t-test (*P*<0.01), the expression levels of 8, 111, and 18 genes increased by more than 2-fold in Δ*mutS*, Δ*mutL*, and Δ*mutS2* cells, respectively (Fig. 3A and Supplementary Tables S2, S3, S4). It should be mentioned that disruption of *mutL* by reversed *HTK* cassette did not perturb the expression of *mutS* that locates in the upstream of *mutL* in the same operon (Supplementary Table S5). Seven genes *ttha0674*, *ttha0675*, *ttha0676*, *ttha0677*, *ttha0678*, *ttha0679*, and *ttha0680* were suggested to be up-regulated in all of the three disruptant strains (Fig. 3A). These genes are coded in the same operon (Fig. 3B). It has been clarified that insertion of *HTK* cassette does not influence the up-regulation of these genes (GEO accession No. GSE7166, 10369, and GSE19521). As shown in Fig. 3C and D, they are the genes for vitamin B_1_ (thiamine) biosynthesis except for *ttha0679*. The amino acid sequence of TTHA0679 showed similarity to those of major facilitator superfamily transporters. It has been known that yeast vitamin B_1_ transporter THI7 belongs to this superfamily, implying that TTHA0679 is also a vitamin B_1_ transporter. The up-regulations of these genes in the disruptant cells were also confirmed by RT-PCR (Fig. 3E). Thus, vitamin B_1_ biosynthesis genes were commonly stimulated in Δ*mutS*, Δ*mutL*, and Δ*mutS2* cells. It is also suggested that a putative vitamin B_1_-transporter gene (*ttha1807*) was up-regulated in Δ*mutS* and Δ*mutL* cells (Supplementary Tables S2 and S3).

**Figure 4:**
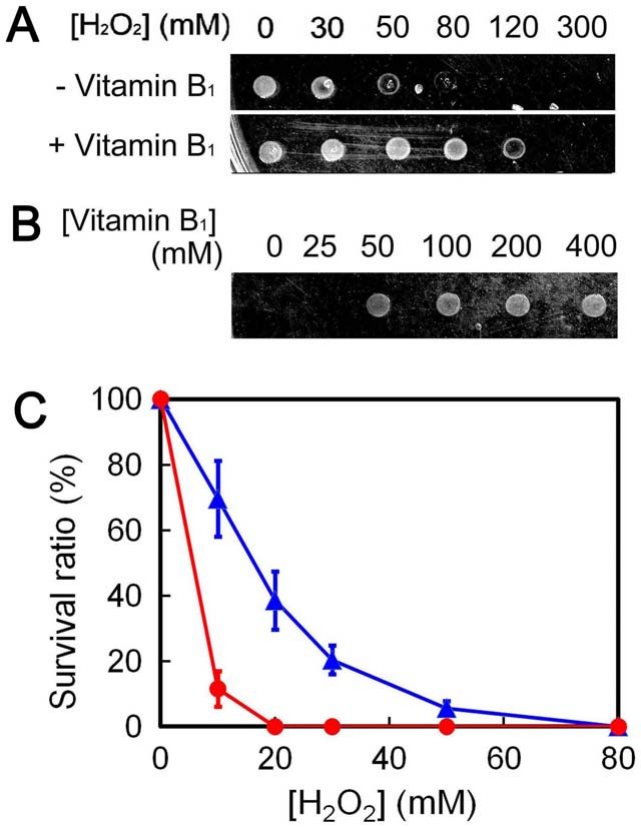
Vitamin B_1_ positively affects the survival of *T. thermophilus* HB8 under oxidative stress. (A) The effect of 0 (*upper panel*) or 50 (*lower panel*) mM vitamin B_1_ on the tolerance of *T. thermophilus* HB8 wild-type strain to H_2_O_2_. Cells were incubated with various concentrations of H_2_O_2_ and then spotted onto plates as described under *Methods*. The concentrations of H_2_O_2_ are indicated at the top of the panel. (B) The vitamin B_1_ dose dependence of the H_2_O_2_ sensitivity. *T. thermophilus* HB8 wild-type strain was incubated in broth containing 50 mM H_2_O_2_ and the indicated concentrations of vitamin B_1_. (C) Sensitivity of Δ*ttha0675* cells to H_2_O_2_. Wild-type and Δ*ttha0675* strains were incubated in the broth containing 0, 10, 20, 30, 50, and 80 mM H_2_O_2_. After treatment with H_2_O_2_, cells were spread onto plates and incubated at 70°C for 24 h. The survival ratios of the wild-type (triangles) and Δ*ttha0675* (circles) strains were estimated based on the numbers of colonies on the plates and plotted against H_2_O_2_ concentration. Bars indicate standard deviations.

Previous studies suggested the ROS-scavenging ability of vitamin B_1_ [50], [51], [52], [53] and the oxidative stress-induced increase in the intracellular level of vitamin B_1_ level in *Arabidopsis thaliana* [54]. In order to account for the antioxidant effects of vitamin B_1_, Gibson and Blass proposed two chemical reaction pathways which involve opening of thiazole ring or formation of tricyclic thiamine [53]. Then, we examined whether or not vitamin B_1_ has this effect on the survival of *T. thermophilus* HB8 cells under H_2_O_2_-induced stress. As shown in Fig. 4A and B, the addition of vitamin B_1_ to the medium resulted in reduced sensitivity to H_2_O_2_. *T. thermophilus* HB8 has genes for ABC transporter subunits (*ttha1807* and *ttha1808*), whose amino acid sequences are highly similar to those of vitamin B_1_ transporter subunits. Therefore, it is expected that *T. thermophilus* HB8 can uptake exogenous vitamin B_1_ into cells. Alternatively, it is also possible that vitamin B_1_ detoxified H_2_O_2_ in the medium prior entering the cells, since the time interval between vitamin B_1_ addition and H_2_O_2_ addition was 5 min. We also examined the effect of *ttha0675*-knockout on the tolerance to H_2_O_2_-induced stress. Since *ttha0675*-disrupted cells (Δ*ttha0675*) showed an obvious delay in their growth even in rich medium, we measured the survival ratio to evaluate the tolerance to H_2_O_2_-induced stress. As a result, we observed a significant decrease in the survival ratio of the Δ*ttha0675* strain compared with the wild-type strain (Fig. 4C). This finding suggests that vitamin B_1_ can serve as an antioxidant against ROS also in *T. thermophilus* HB8.

### *mutS*, *mutL*, and *mutS2*-lacking strains exhibited increased mutation frequencies under oxidative stress

**Figure 5:**
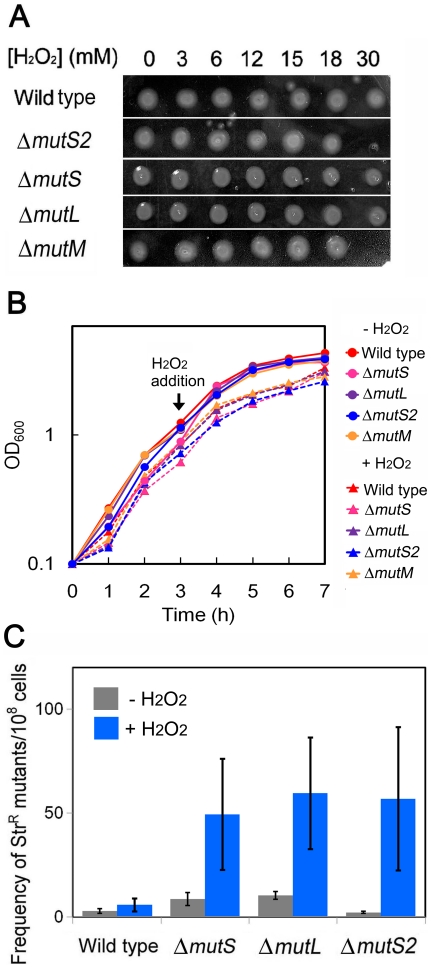
Effect of gene disruption on the tolerance to H_2_O_2_ and the rate of H_2_O_2_-induced mutagenesis. (A) Sensitivity to H_2_O_2_. The wild-type, Δ*mutS2,* Δ*mutS*, Δ*mutL*, and Δ*mutM* strains of *T. thermophilus* HB8 were incubated in medium containing the indicated concentrations of H_2_O_2_. After incubation with H_2_O_2_, cells were spotted onto plates. (B) Effect of 10 mM H_2_O_2_ on growth curves of the wild-type (*red*), Δ*mutS* (*pink*), Δ*mutL* (*purple*), Δ*mutS2* (*blue*), and Δ*mutM* (*orange*) strains of *T. thermophilus* HB8. Precultured cells were inoculated to 5 ml of medium to an OD_600_ value of 0.10. After incubation at 70°C for 3 h, 50 µl of 0 (*circles*) or 1 M (*triangles*) H_2_O_2_ was added. (C) Rate of H_2_O_2_-induced mutagenesis. The wild-type, Δ*mutS,* Δ*mutL*, and Δ*mutS2* strains of *T. thermophilus* HB8 were incubated in medium containing 0 or 10 mM H_2_O_2_ for 30 min. After incubation, cells were spread onto plates containing 0 or 50 µg/ml streptomycin. Frequency of streptomycin-resistant mutants per 10^8^ cells was calculated from the numbers of colonies formed on the streptomycin-containing and drug-free plates. Bars indicate standard deviations.

The induction of vitamin B_1_ biosynthesis implies an increased level of oxidative stress and/or damage in the Δ*mutS*, Δ*mutL*, and Δ*mutS2* strains. Thus, we compared the H_2_O_2_-sensitivities of the three disruptant strains with that of the wild-type strain. The disruptants showed no remarkable increase in the sensitivity to H_2_O_2_ (below 30 mM), although the Δ*mutS2* strain exhibited a slightly increased sensitivity to 30 mM H_2_O_2_ to the same extent as the strain lacking *mutM* that encodes the base-excision repair glycosylase (Fig. 5A and B). On the other hand, the Δ*mutS*, Δ*mutL*, and Δ*mutS2* strains, under oxidative stress caused by 10 mM H_2_O_2_, showed significantly higher mutation frequencies than the wild-type strain (Fig. 5C), suggesting the involvement of these genes in the repair of mutagenic oxidative DNA damage such as 8OG and 5-formyluracil. It should be mentioned that streptomycine-resistance-based measurement of mutation frequency performed here detects the frequency of the single-base substitutions including AT-CG transversion and AT-GC transition mutations [55] which can be generated by 8OG and 5-formyluracil, respectively [11], [56], [57]. Our DNA microarray experiments suggested that transcriptions of *ttha1934* and *tthb071* were also up-regulated in Δ*mutL*, and Δ*mutS2* cells, respectively (Supplementary Tables S3 and S4). These genes encode proteins whose amino acid sequences exhibit significant similarity to those of apurinic/apyrimidinic endonucleases. Apurinic/apyrimidinic endonucleases are generally required for base-excision repair system to process the abasic sites generated by DNA glycosylases [58]. Therefore, it can be expected that proteins encoded by *ttha1934* and *tthb071* are also involved in the repair of oxidative DNA damage.

## Discussion

DNA microarray experiments demonstrated that transcription of the vitamin B_1_ biosynthesis operon was commonly up-regulated in Δ*mutS*, Δ*mutL*, and Δ*mutS2* cells (Fig. 3). It has been well established that, in many bacteria, the vitamin B_1_ biosynthesis operon is regulated by thiamin pyrophosphate-binding riboswitch at the translational level [59], [60], and the riboswitch-coding sequence is called the *thi* element [61]. However, *T. thermophilus* has no *thi* element in the 5′-untranslated region of the vitamin B_1_ biosynthesis operon [61]. Therefore, it had been expected that the expression of the operon is regulated at the transcriptional level, and this notion was strongly supported by our transcriptomic analyses.

The activation of vitamin B_1_ biosynthesis was also observed when *T. thermophilus* HB8 cells were cultured in the medium containing 10 mM H_2_O_2_ [62]. It has been reported that vitamin B_1_ can serve as a direct ROS scavenger [51], [52] in addition to its role as a co-factor for several reductases (the coenzyme form of vitamin B_1_ is thiamine pyrophosphate). In good agreement with this, disruption of a vitamin B_1_-biosynthesis gene (*ttha0675*) resulted in drastic increase in the H_2_O_2_-sensitivity (Fig. 4C) and the addition of vitamin B_1_ to the medium enhanced the tolerance to H_2_O_2_ (Fig. 4A and B).

Although *T. thermophilus* HB8 has genes encoding peroxiredoxin (TTHA1300), Mn catalase (TTHA0122), and heme peroxidase (TTHA1714), which potentially detoxify H_2_O_2_, we did not observe the activation of these genes in Δ*mutS*, Δ*mutL*, and Δ*mutS2* cells. Vitamin B_1_ has been reported to show scavenging activity against hydroxyl radical that is derived from H_2_O_2_ through Fenton reaction in the cell and directly attacks DNA molecules [51]. For oxidatively damaged cells, it might be more effective to detoxify hydroxyl radicals than to detoxify H_2_O_2_.

It can be speculated that the increased production of thiamine is utilized as a coenzyme for various enzymes. However, our microarray analysis did not detect the activation of the gene for thiamine phosphate kinase (TTHA0424), therefore, we believe that the observed activation of thiamine biosynthesis genes does not result in the activation of thiamine pyrophosphate-requiring enzymes.

Since the stimulation of biosynthesis of an ROS-scavenging molecule was observed, it can be thought that there was a significant increase in oxidative stress and/or damage in Δ*mutS*, Δ*mutL*, and Δ*mutS2* cells. Considering the previously reported molecular functions of MutS, MutL, and MutS2, these proteins are expected to participate in the repair of oxidative DNA damage but not in detoxifying ROS. This hypothesis was supported by our finding that disruption of *mutS*, *mutL*, or *mutS2* did not cause a drastic decrease in the survival ratio under H_2_O_2_ (below 30 mM)-induced stress but in a significant increase in the rate of 10 mM H_2_O_2_-induced mutagenesis (Fig. 5A–C). In addition, no up-regulation was observed for the transcriptional regulator SdrP, whose expression level is greatly up-regulated in response to the oxidative stress [40] (Supplementary Tables S2, S3, S4), suggesting that there was elevation of DNA damages but no elevation of the oxidative stress in Δ*mutS*, Δ*mutL*, and Δ*mutS2* cells. The oxidative DNA damage-dependent alteration of the genetic information, if accumulated, should affect not only the mutant frequency but also the survival of the cell. Therefore, it would be possible that higher concentrations of H_2_O_2_ decrease the survival ratio of Δ*mutS*, Δ*mutL*, and Δ*mutS2* cells.

The results of DNA microarray and phenotypic analyses implied the cooperative function of MutS, MutL, and MutS2 in repair of oxidative DNA damages, which is consistent with our finding of *in vivo* interactions of MutS2 with MutS or MutL (Fig. 2). Interaction between MutS2 and MutS is reasonable when we remember that MutS2 retains the region homologous to the dimerization domain of MutS [31]. Although there has been no report about the heterodimerization of bacterial MutS homolgoues, it is known that eukaryotic MutS homologues form heterodimers (Fig. 1A) [63], [64]. The observed interaction between MutS2 and MutS might indicate the existence of the heterodimer of bacterial MutS homolgoues. As to the MutS2-MutL interaction, there might be an analogy with the well-characterized interaction between MutS and MutL. Recently, it was reported that the connector domain of MutS comprises the interface with MutL [65]. Although MutS2 seems to retain the region corresponding to the connector domain of MutS [31], the poor sequence conservation in the region prevents us from identifying the MutL-interacting residues in MutS2. The repair machinery including these interactions should be investigated in future studies.

Disruption of *mutS*, *mutL*, or *mutS2* had no remarkable effect on the survival of *T. thermophilus* HB8 but did on the rate of mutagenesis under H_2_O_2_-induced oxidative stress (Fig. 5). Therefore, it is expected that these genes are responsible for the repair of mutagenic DNA damage such as 8OG, 5-formyluracil, and 5-hydroxymethyluracil rather than fatal DNA damages such as thymine glycol which blocks DNA synthesis [66]. It should be mentioned that oxidative stress has an enhancing effect on the deamination of exocyclic amino groups of bases in DNA, which generates mutagenic bases such as uracil and hypoxanthine [3]. Uracil and hypoxanthine can be yielded through the deaminations of cytosine and adenine, which cause GC-AT and AT-GC transition mutations, respectively [67]. These mutagenic bases are also potential candidate for the substrates of MutS, MutL, and MutS2.

MutS2 homologues are usually present in MutS- and MutL-containing species [68], [69]. The cooperation of MutS2 with MutS and MutL might be universal for these species. The exceptions are the several pathogens among ε-Proteobacteria such as *H. pylori*, which has MutS2 but not MutS and MutL [27], [28]. In these pathogens, DNA repair activities are expected to be significantly reduced. The increase in the mutation frequency caused by the loss of the DNA repair activity may be an advantage for these pathogens that need to adapt to a frequently changing environment. Interestingly, *H. pylori* MutS2 shows specific binding activity toward 8OG-containing DNA [30], while *T. thermophilus* MutS2 did not exhibit such specificity (data not shown). It may be possible that MutS2 homodimer and a MutS-MutS2 complex are responsible for the recognition of oxidatively damaged DNA in *mutS*-lacking and *mutS*-containing species, respectively. There is, of course, another possibility that a protein other than MutS homologues recognizes the damaged DNA and MutS-MutS2 supports it.

DNA microarray analyses revealed that the number of up-regulated genes in Δ*mutL* was more than tenfold greater than seen in either Δ*mutS* or Δ*mutS2* cells (Supplementary Tables. S2, S3, S4). This finding may indicate that MutL has additional functions besides the repair of mismatched bases and oxidative damage. Remarkably, Δ*mutL*-specific up-regulation was observed for genes *tthb148-152 tthb178*, *tthb187*, and *tthb190-194* (Supplementary Table S3), which are under the control of a transcriptional regulator, cAMP-dependent protein (CRP) [39]. These genes are characteristic of the clustered regularly interspaced short palindromic repeat (CRISPR), and so called CRISPR-associated (*cas)* genes. The *cas* genes have been implicated as components of a host defense system against invading foreign replicons [70]. Recently, it was verified that CRP up-regulates the *cas* genes upon phage infection in *T. thermophilus* HB8 [71]. Hence, we could speculate that cells lacking the *mutL* gene need to be ready for the attack by the foreign replicon. It should be noted that, in Δ*mutL* cells, the expression of the type II restriction enzyme *Tth*HB8I (*ttha1548*) was also induced (Supplementary Table S3), while that of DNA recombinase RecA was suppressed (Supplementary Table S5). Since MMR is also known to function in the protection of cells from invading foreign DNAs by preventing homeologous recombination, inactivation of *mutL* may increase the opportunity to be attacked by the incoming DNA. However, DNA microarray analyses did not detect any increase in the expression of the *cas* genes or restriction enzymes in cells lacking another MMR gene, *mutS* (Supplementary Table S2). These results imply that MutL is responsible for an additional role other than DNA repair.

**Figure 6:**
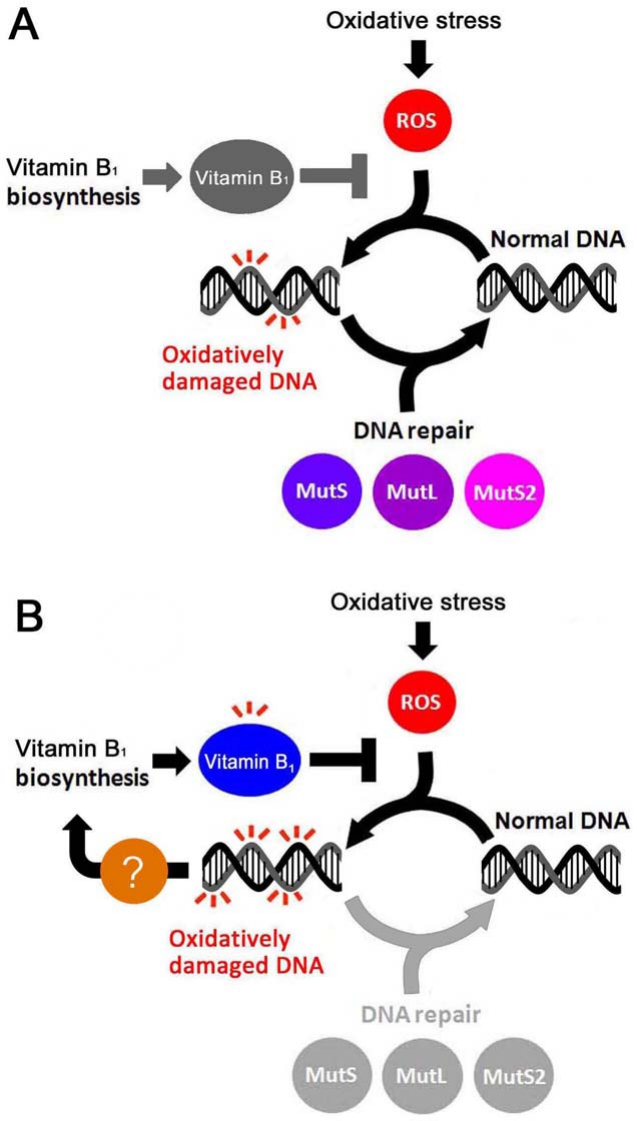
Inactivation of DNA repair genes leads to the induction of vitamin B_1_ biosynthesis. Extracellular oxidative stress and intracellular redox metabolism generate ROS, which can attack DNA to yield oxidatively damaged DNA. (A) In the wild-type strain, oxidatively damaged DNA is repaired by DNA repair enzymes including MutS, MutL, and MutS2. (B) In the Δ*mutS*, Δ*mutL*, and Δ*mutS2* strains, the genes for vitamin B_1_ biosynthesis are activated to prevent the accumulation of oxidative damage in DNA via an unknown mechanism.

This study clarified that inactivation of DNA repair enzymes leads to the activation of a ROS-detoxifying mechanism (Fig. 6). In other words, our results suggest that a ROS-detoxifying system can be regulated corresponding to the intracellular level of oxidatively damaged DNA or the absence of DNA repair enzymes. Although a previous study revealed the intracellular ROS level-dependent transcriptional regulation of the ROS-detoxifying system [54], to the best of our knowledge, this is the first report that the loss of DNA repair functions or the accumulation of DNA damage can stimulate the expression of the ROS-detoxifying system. It remains to be investigated how cells sense the accumulation of oxidative DNA damage or absence of DNA repair enzymes, and signal the information to the regulation machinery of the vitamin B_1_-dependent ROS-detoxifying system. Since there was no candidate for the transcriptional regulator whose expression level was up- or down-regulated upon disruption of *mutS*, *mutL*, or *mutS2* gene (data not shown), it can be speculated that a constitutively expressed transcriptional regulator is responsible for the regulation of the vitamin B_1_-dependent ROS-detoxifying system upon inactivation of DNA repair enzymes.

## Supporting Information

Table S1 Primers used in RT-PCR experiments.
(DOC)

Table S2 Genes up-regulated in Δ*mutS* cells.
(DOC)

Table S3 Genes up-regulated in Δ*mutL* cells.
(DOC)

Table S4 Genes up-regulated in Δ*mutS2* cells.
(DOC)

Table S5 Genes down-regulated in Δ*mutL* cells.
(DOC)
