# Supplementary material for: Inactivation of the DNA Repair Genes mutS, mutL or the Anti-Recombination Gene mutS2 Leads to Activation of Vitamin B1 Biosynthesis Genes
Source: PLoS One. 2011 Apr 28;6(4):e19053. doi: 10.1371/journal.pone.0019053 (PMC3084264; doi:10.1371/journal.pone.0019053)
Supplement: Table S1 — Primers used in RT-PCR experiments. (DOC) [file pone.0019053.s001.doc]

Table S1. Primers used in RT-PCR experiments.

| Primer name | Sequence (5’-3’) |
| --- | --- |
| *ttha0674*-Forward | GGAAGGCTCTACCTGGTGGTGACCCCAAGG |
| *ttha0674*-Reverse | CAAAGAAGCGCCGCGCCTCCTCGGGG |
| *ttha0675*-Forward | GTGTGGCTTAACGGGGAGCCCAGGCCCTTG |
| *ttha0675*-Reverse | ACCACGTCCCCGTCCCGCAAGGGGCGGTCC |
| *ttha0676*-Forward | CAAGTACGAGGACTTCGGGGTGATGCGGG |
| *ttha0676*-Reverse | CTCCGCCGCCTTCAGGGTCTCCAGGGGGTC |
| *ttha0677*-Forward | CGCCGCCTACGAGCTCGCCAAGCGGG |
| *ttha0677*-Reverse | AACCCCCCGGGGAAGCGCCTCGCCCCCAAG |
| *ttha0678*-Forward | CCTACGTGGCCGAGAAGGAAGGGGTTTCCC |
| *ttha0678*-Reverse | TGTCCGCCCCGTACTGGATGGCCACCCTGG |
| *ttha0679*-Forward | CCCGTCTGGAACGCCTGCCCTTGGGCAGG |
| *ttha0679*-Reverse | GGCGGTGAGGAGGCTTCCCAGCCCAGCGAG |
| *ttha0680*-Forward | CATCGCGGGCTCCGACTCGGGGGGCG |
| *ttha0680*-Reverse | AAGCCTCTCCTTGAGGGCGGCCGCCGCC |
