# Supplementary material for: Inactivation of the DNA Repair Genes mutS, mutL or the Anti-Recombination Gene mutS2 Leads to Activation of Vitamin B1 Biosynthesis Genes
Source: PLoS One. 2011 Apr 28;6(4):e19053. doi: 10.1371/journal.pone.0019053 (PMC3084264; doi:10.1371/journal.pone.0019053)
Supplement: Table S2 — Genes up-regulated in ΔmutS cells. (DOC) [file pone.0019053.s002.doc]

Table S2. Genes up-regulated in Δ*mutS* cells.

| Gene name | Expression*^1^ | *P*-value | Annotation for product | COG code*^2^ |
| --- | --- | --- | --- | --- |
| *ttha0676* | 6.5 | 0.00028 | Thiazole biosynthesis protein ThiG | H |
| *ttha0678* | 5.4 | 0.00033 | Thiamine biosynthesis protein ThiC | H |
| *ttha0677* | 4.6 | 0.00037 | Thiamine biosynthesis oxidoreductase ThiO | E |
| *ttha0679* | 4.5 | 0.00087 | Putative transport protein | GEPR |
| *ttha0674* | 4.2 | 0.00016 | Thiamine-phosphate pyrophosphorylase | H |
| *ttha0680* | 3.0 | 0.00035 | Phosphomethylpyrimidine kinase ThiD | H |
| *ttha0454* | 2.0 | 0.000080 | ABC transporter, permease protein | P |
| *ttha1807* | 2.0 | 0.00013 | ABC transporter, permease protein | P |

*^1^Normalized intensity of the Δ*mutS* strain relative to that of the wild-type strain.

*^2^The descriptions for each COG code are Translation (L), Transcription (K), Replication, recombination, and repair (L), Chromatin structure and dynamics (B), Cell cycle control, mitosis, and meiosis (D), Defense mechanisms (V), Signal transduction mechanisms (T), Cell wall/membrane biogenesis (M), Cell motility (N), Cytoskeleton (Z), Intracellular trafficking and secretion (U), Posttranslational modification, protein turnover, and chaperones (O), Energy production and conversion (C), Carbohydrate transport metabolism (G), Amino acid transport and metabolism (E), Nucleotide transport and metabolism (F), Coenzyme transport and metabolism (H), Lipid transport and metabolism (I), Inorganic ion transport and metabolism (P), Secondary metabolite biosynthesis, transport, and catabolism (Q), General function prediction only (R), Function unknown (S), and Not in COGs (-).
