# Supplementary material for: Inactivation of the DNA Repair Genes mutS, mutL or the Anti-Recombination Gene mutS2 Leads to Activation of Vitamin B1 Biosynthesis Genes
Source: PLoS One. 2011 Apr 28;6(4):e19053. doi: 10.1371/journal.pone.0019053 (PMC3084264; doi:10.1371/journal.pone.0019053)
Supplement: Table S3 — Genes up-regulated in ΔmutL cells. (DOC) [file pone.0019053.s003.doc]

Table S3. Genes up-regulated in Δ*mutL* cells.

| Gene name | Expression* | *P*-value | Annotation for product | COG code |
| --- | --- | --- | --- | --- |
| *tthb156* | 31 | 0.000015 | Hypothetical protein | - |
| *tthb158* | 12 | 0.0018 | Hypothetical protein | - |
| *tthb157* | 12 | 0.000056 | Hypothetical protein | - |
| *tthb178* | 6.6 | 0.000015 | Conserved hypothetical protein | L |
| *ttha0679* | 6.4 | 0.00019 | Putative transport protein | GEPR |
| *ttha0674* | 5.7 | 0.000014 | Thiamine-phosphate pyrophosphorylase | H |
| *tthb186* | 5.7 | 0.0067 | Conserved hypothetical protein | K |
| *ttha0678* | 5.7 | 0.00095 | Thiamine biosynthesis protein ThiC | H |
| *ttha0676* | 5.5 | 0.000026 | Thiazole biosynthesis protein ThiG | H |
| *ttha0677* | 5.3 | 0.00076 | Thiamine biosynthesis oxidoreductase ThiO | E |
| *tthb187* | 5.0 | 0.00018 | Conserved hypothetical protein | R |
| *tthb165* | 4.5 | 0.00016 | Conserved hypothetical protein | L |
| *ttha1321* | 4.5 | 0.000016 | Formyltetrahydrofolate deformylase | F |
| *ttha1874* | 4.3 | 0.00035 | Hypothetical protein | - |
| *ttha1349* | 4.0 | 0.00039 | DNA-binding protein HU | L |
| *tthb164* | 3.8 | 0.00021 | Conserved hypothetical protein | L |
| *ttha0963* | 3.7 | 0.0032 | ABC tranpsorter, ATP binding protein related protein | E |
| *tthb162* | 3.6 | 0.0021 | Conserved hypothetical protein | L |
| *ttha1221* | 3.6 | 0.00011 | Pilin, type IV, putative | - |
| *tthb163* | 3.4 | 0.0018 | Conserved hypothetical protein | L |
| *ttha0680* | 3.4 | 0.00058 | Phosphomethylpyrimidine kinase ThiD | H |
| *tthb050* | 3.4 | 0.0017 | Putative nickel transporter | P |
| *ttha1893* | 3.4 | 0.00018 | S-layer protein precursor P100 protein | D |
| *tthb160* | 3.4 | 0.0035 | Conserved hypothetical protein | R |
| *ttha1873* | 3.2 | 0.00012 | Hypothetical protein | C |
| *ttha0243* | 3.2 | 0.00030 | 30S ribosomal protein S18 | J |
| *tthb192* | 3.0 | 0.00048 | Conserved hypothetical protein | - |
| *ttha0418* | 3.0 | 0.00022 | 50S ribosomal protein L32 | J |
| *ttha1479* | 3.0 | 0.0014 | Conserved hypothetical protein | - |
| *tthb214* | 2.9 | 0.0027 | Hypothetical proteih | - |
| *tthb190* | 2.9 | 0.00021 | Conserved hypothetical protein | - |
| *tthb017* | 2.9 | 0.0030 | Putative medium-chain acyl-CoA ligase | IQ |
| *ttha0970* | 2.9 | 0.00023 | Phenylacetic acid degradation protein PaaC | S |
| *tthb194* | 2.8 | 0.00053 | Conserved hypothetical protein | - |
| *ttha0244* | 2.8 | 0.00027 | Single-stranded DNA binding protein | L |
| *tthb013* | 2.8 | 0.0016 | Hypothetical protein | - |
| *ttha0966* | 2.8 | 0.0016 | Phenylacetyl-CoA ligase | H |
| *ttha0386* | 2.8 | 0.00011 | Sec-independent protein translocase protein TatA | U |
| *ttha0245* | 2.7 | 0.00010 | 30S ribosomal protein S6 | J |
| *tthb191* | 2.7 | 0.00043 | Conserved hypothetical protein | L |
| *ttha1909* | 2.7 | 0.00019 | Conserved hypothetical protein | S |
| *ttha0964* | 2.7 | 0.00083 | ABC transporter ATP-binding protein related protein | E |
| *tthb037* | 2.7 | 0.0073 | Hypothetical protein | T |
| *tthb022* | 2.6 | 0.0057 | Putative acyl-CoA dehydrogenase | I |
| *ttha0268* | 2.6 | 0.0018 | Hypothetical protein | - |
| *ttha1634* | 2.6 | 0.00021 | Peptide ABC transporter, peptide-binding protein | E |
| *ttha0850* | 2.6 | 0.00040 | Stage V sporulation protein S related protein | S |
| *ttha0285* | 2.6 | 0.0081 | Conserved hypothetical protein | - |
| *ttha0027* | 2.6 | 0.000075 | Probable potassium channel, beta subunit | C |
| *ttha0272* | 2.6 | 0.0020 | 10 kDa chaperonin (Protein Cpn10) (GroES protein) | O |
| *tthb054* | 2.6 | 0.00066 | Precorrin-2 methylase | H |
| *ttha0944* | 2.5 | 0.0029 | Hypothetical protein | - |
| *tthb193* | 2.5 | 0.000035 | Conserved hypothetical protein | L |
| *ttha0945* | 2.5 | 0.0013 | Hypothetical protein | - |
| *tthb180* | 2.5 | 0.00060 | Conserved hypothetical protein | S |
| *ttha0987* | 2.5 | 0.0046 | Beta-ketoadipyl CoA thiolase | I |
| *ttha1578* | 2.4 | 0.0044 | 1-pyrroline-5-carboxylate dehydrogenase | C |
| *ttha0959* | 2.4 | 0.0089 | 5-carboxymethyl-2-hydroxymuconate semialdehyde dehydrogenase | C |
| *ttha0233* | 2.4 | 0.00019 | Pyruvate dehydrogenase complex, dihydrolipoamide dehydrogenase E3 component | C |
| *tthb148* | 2.4 | 0.00043 | Hypothetical protein | - |
| *ttha1604* | 2.4 | 0.00014 | Conserved hypothetical protein | - |
| *tthb021* | 2.4 | 0.0023 | Conserved hypothetical protein | V |
| *tthb052* | 2.4 | 0.00037 | Cobalamin biosynthesis precorrin isomerase | H |
| *ttha1560* | 2.4 | 0.0011 | DNA pantothenate metabolism flavoprotein | H |
| *ttha1807* | 2.4 | 0.000011 | ABC transporter, permease protein, CysTW family | P |
| *tthb149* | 2.3 | 0.0031 | Conserved hypothetical protein | L |
| *tthb152* | 2.3 | 0.00014 | Conserved hypothetical protein | L |
| *ttha0271* | 2.3 | 0.0015 | 60 kDa chaperonin (Protein Cpn60) (GroEL protein) | O |
| *ttha1484* | 2.3 | 0.00026 | Heat shock protein, HSP20 family | O |
| *ttha1020* | 2.3 | 0.0012 | Conserved hypothetical protein | KL |
| *ttha0958* | 2.3 | 0.0011 | 2-hydroxyhepta-2,4-diene-1,7-dioate isomerase 5-carboxymethyl-2-oxo-hex-3-ene-1,7-dioate decarboxylase | Q |
| *ttha1934* | 2.3 | 0.000035 | Conserved hypothetical protein | G |
| *ttha1325* | 2.3 | 0.0039 | Putaitve sulfite oxidase | R |
| *ttha0553* | 2.3 | 0.000071 | Ribosomal protein L20 | J |
| *ttha0213* | 2.3 | 0.0035 | Hypothetical protein | - |
| *tthb142* | 2.3 | 0.00033 | Glycerol kinase | C |
| *ttha1465* | 2.3 | 0.0022 | 50S ribosomal protein L13 | J |
| *ttha0969* | 2.3 | 0.0013 | Phenylacetic acid degradation protein PaaD | R |
| *ttha1147* | 2.2 | 0.00020 | Hypothetical protein | - |
| *ttha0999* | 2.2 | 0.0022 | Hypothetical protein | T |
| *tthb150* | 2.2 | 0.00029 | Conserved hypothetical protein | - |
| *tthb028* | 2.2 | 0.0016 | Hypothetical protein | C |
| *ttha1166* | 2.2 | 0.00054 | Hypothetical protein | - |
| *ttha1612* | 2.2 | 0.00043 | Putative hydrolase | R |
| *ttha1662* | 2.2 | 0.0027 | Conserved hypothetical protein | - |
| *ttha1697* | 2.2 | 0.0039 | 30S ribosomal protein S12 | J |
| *ttha0972* | 2.2 | 0.0020 | Phenylacetic acid degradation protein PaaA | S |
| *ttha1326* | 2.1 | 0.0070 | Cytochrome c-552 like protein | C |
| *ttha1652* | 2.1 | 0.0017 | Maltose ABC transporter, periplasmic maltose-binding protein | G |
| *ttha0346* | 2.1 | 0.00036 | Peptidyl-prolyl cis-trans isomerase | O |
| *ttha0315* | 2.1 | 0.0045 | Conserved hypothetical protein | R |
| *ttha0846* | 2.1 | 0.0084 | Metallo-beta-lactamase family protein | R |
| *ttha0201* | 2.1 | 0.0051 | Mg^2+^ chelatase family protein | O |
| *ttha1543* | 2.1 | 0.00065 | Hypothetical protein | - |
| *ttha1656* | 2.1 | 0.0022 | Hypothetical protein | - |
| *ttha0379* | 2.1 | 0.00027 | Sugar ABC transporter, periplasmic sugar-binding protein | G |
| *ttha1335* | 2.1 | 0.00024 | Branched-chain amino acid ABC transporter, ATP-binding protein | E |
| *tthb051* | 2.1 | 0.0050 | Cobalamin biosynthetic protein | H |
| *ttha1464* | 2.1 | 0.00045 | 30S ribosomal protein S9 | J |
| *ttha0175* | 2.1 | 0.00035 | Cold shock protein | K |
| *ttha0800* | 2.1 | 0.0030 | Chorismate mutase, putative | E |
| *ttha0286* | 2.0 | 0.0071 | Serine protease, subtilase family | O |
| *ttha0196* | 2.0 | 0.0077 | ABC transporter, periplasmic binding protein | Q |
| *ttha0209* | 2.0 | 0.00022 | 50S ribosomal protein L10 | J |
| *ttha1584* | 2.0 | 0.000083 | Type II restriction enzyme *Tth*HB8I | - |
| *ttha0961* | 2.0 | 0.0088 | Conserved hypothetical protein | R |
| *ttha1259* | 2.0 | 0.0010 | Adenylate cyclase related protein | T |
| *ttha1966* | 2.0 | 0.00023 | Hypothetical protein | - |
| *ttha1732* | 2.0 | 0.0012 | Conserved hypothetical protein | S |

*Normalized intensity of the Δ*mutL* strain relative to that of the wild-type strain.
