# Supplementary material for: Inactivation of the DNA Repair Genes mutS, mutL or the Anti-Recombination Gene mutS2 Leads to Activation of Vitamin B1 Biosynthesis Genes
Source: PLoS One. 2011 Apr 28;6(4):e19053. doi: 10.1371/journal.pone.0019053 (PMC3084264; doi:10.1371/journal.pone.0019053)
Supplement: Table S4 — Genes up-regulated in ΔmutS2 cells. (DOC) [file pone.0019053.s004.doc]

Table S4. Genes up-regulated in Δ*mutS2* cells.

| Gene name | Expression* | *P*-value | Annotation for product | COG code |
| --- | --- | --- | --- | --- |
| *ttha0679* | 4.6 | 0.000049 | Putative transport protein | GEPR |
| *ttha0676* | 4.2 | 0.00011 | Thiazole biosynthesis protein ThiG | H |
| *ttha0677* | 3.9 | 0.00046 | Thiamine biosynthesis oxidoreductase ThiO | E |
| *ttha0678* | 3.9 | 0.00042 | Thiamine biosynthesis protein ThiC | H |
| *ttha0680* | 3.4 | 0.00042 | Phosphomethylpyrimidine kinase ThiD | H |
| *tthb089* | 3.2 | 0.0010 | Conserved hypothetical protein | - |
| *tthb070* | 3.2 | 0.000015 | Survival protein SurE | R |
| *ttha0674* | 3.1 | 0.00011 | Thiamine-phosphate pyrophosphorylase | H |
| *ttha0968* | 3.1 | 0.000099 | Phenylacetic acid degradation protein PaaZ | CI |
| *ttha0969* | 3.0 | 0.00030 | Phenylacetic acid degradation protein PaaD | R |
| *tthb071* | 2.6 | 0.0000043 | Conserved hypothetical protein | G |
| *ttha0957* | 2.6 | 0.000015 | Dihydrodipicolinate synthase | EM |
| *ttha0580* | 2.3 | 0.00026 | Hypothetical protein | - |
| *tthb067* | 2.2 | 0.00032 | Alkaline phosphatase | P |
| *ttha1110* | 2.1 | 0.0072 | Conserved hypothetical protein | M |
| *ttha0958* | 2.1 | 0.00015 | 2-hydroxyhepta-2,4-diene-1,7-dioate isomerase | Q |
| *ttha0959* | 2.1 | 0.000039 | 5-carboxymethyl-2-hydroxymuconate semialdehyde dehydrogenase | C |
| *ttha0144* | 2.0 | 0.00028 | Sensor histidine kinase | T |

*Normalized intensity of the Δ*mutS2* strain relative to that of the wild-type strain.
