# Supplementary material for: Inactivation of the DNA Repair Genes mutS, mutL or the Anti-Recombination Gene mutS2 Leads to Activation of Vitamin B1 Biosynthesis Genes
Source: PLoS One. 2011 Apr 28;6(4):e19053. doi: 10.1371/journal.pone.0019053 (PMC3084264; doi:10.1371/journal.pone.0019053)
Supplement: Table S5 — Genes down-regulated in ΔmutL cells. (DOC) [file pone.0019053.s005.doc]

Table S5. Genes down-regulated in Δ*mutL* cells.

| Gene name | Expression* | *P*-value | Annotation for product | COG code |
| --- | --- | --- | --- | --- |
| *ttha0605* | 0.50 | 0.00030 | 4-hydroxybenzoate octaprenyltransferase | H |
| *ttha1944* | 0.49 | 0.0012 | Conserved hypothetical protein | S |
| *ttha0486* | 0.48 | 0.0016 | Conserved hypothetical protein | C |
| *ttha0331* | 0.48 | 0.0013 | Histidinol phosphatase | ER |
| *ttha1066* | 0.48 | 0.0059 | Probable transaldolase | G |
| *ttha0464* | 0.48 | 0.0046 | Hypothetical protein | - |
| *ttha1585* | 0.48 | 0.0090 | Hypothetical protein | - |
| *ttha0497* | 0.47 | 0.0046 | Oxygen-independent coproporphyrinogen III oxidase | H |
| *ttha0869* | 0.47 | 0.00077 | LmbE-related protein | S |
| *ttha0377* | 0.47 | 0.0044 | Sugar ABC transporter, permease protein | G |
| *ttha1199* | 0.46 | 0.025 | Ornithine carbamoyltransferase | E |
| *ttha1429* | 0.46 | 0.0056 | Conserved hypothetical protein | R |
| *ttha0087* | 0.45 | 0.0017 | NADH-quinone oxidoreductase chain 4 | C |
| *ttha0601* | 0.45 | 0.0036 | Uroporphyrinogen decarboxylase | H |
| *tthb218* | 0.45 | 0.019 | ABC transporter, ATP-binding protein | P |
| *ttha0631* | 0.45 | 0.000084 | Heat shock protein HslV | O |
| *ttha0561* | 0.44 | 0.0019 | Outer membrane protein | M |
| *ttha0399* | 0.44 | 0.000040 | Hypothetical protein | - |
| *ttha1818* | 0.43 | 0.0013 | RecA protein (Recombinase A) | L |
| *ttha0373* | 0.42 | 0.00084 | Conserved hypothetical protein | H |
| *ttha0359* | 0.40 | 0.0044 | Cold shock protein, CSD family | K |
| *ttha0725* | 0.40 | 0.016 | Membrane-bound protein LytR | K |
| *ttha0396* | 0.40 | 0.0025 | Conserved hypothetical protein | M |
| *ttha0212* | 0.39 | 0.0018 | Conserved hypothetical protein | S |
| *ttha1173* | 0.39 | 0.0024 | Trk system potassium uptake protein | P |
| *ttha1786* | 0.38 | 0.00017 | Conserved hypothetical protein | - |
| *ttha0805* | 0.37 | 0.019 | Hypothetical protein | - |
| *ttha0091* | 0.36 | 0.0040 | NADH-quinone oxidoreductase chain 8 | C |
| *ttha0440* | 0.36 | 0.0013 | Conserved hypothetical protein | S |
| *ttha0684* | 0.32 | 0.00038 | Probable TolQ-type transport protein | U |
| *ttha0382* | 0.31 | 0.0013 | Conserved hypothetical protein | - |
| *ttha0439* | 0.30 | 0.000057 | ABC transporter, ATP-binding protein | R |
| *ttha1836* | 0.29 | 0.00095 | Isocitrate lyase | C |

*Normalized intensity of the Δ*mutL* strain relative to that of the wild-type strain.
